# Squamous Cell Carcinoma in Combination with a Symbrachydactyly: Initial Management and Long-Term Followup

**DOI:** 10.1155/2014/684130

**Published:** 2014-06-15

**Authors:** Tomas Sanchez, Daniel Walder, Philipp Esenwein

**Affiliations:** Department of Orthopaedic Surgery, Kantonsspital Olten, Baslerstraße 150, 4600 Olten, Switzerland

## Abstract

A 68-year-old female patient presented with a rapidly growing, exulcerating tumor of the left hand in the area of a congenital symbrachydactyly at the digiti II and III. A biopsy of the tumor showed a squamous cell carcinoma. Further workup showed two suspicious axillar enhancements with no evidence of bony infiltration and no further metastasis. An amputation of the second and third ray of the left hand at the metacarpal level and additionally an axillar revision and lymph node dissection were performed and confirmed the suspicion of a squamous cell carcinoma, fortunately without affection of any lymph nodes. After 9 years the patient showed an excellent function of the left hand. Symbrachydactyly malformations and squamous cell carcinoma of the hand are both rare conditions. We could not find a reference that shows a common genetic condition to both and so far this is the first description of a squamous cell carcinoma in the region of a symbrachydactyly. It remains unclear whether our case is a coincidence of two rare independent diseases or there is a pathogenetic link between the malformation and the tumor on a genetic level.

## 1. Introduction

Tumors of the soft tissue and malformations of the hand are both rare conditions [1]. Within the group of soft-tissue tumors, squamous cell carcinoma is most common [2, 3]. Symbrachydactyly is a rare congenital anomaly characterized by limb and digital anomalies. Syndactylias and brachydactylias occur as isolated malformations or in combination with additional dysplasias (aplasia of the major pectoral muscle and ipsilateral mamma, called Poland syndrome [4, 5] or different types of tumors as a part of a syndrome [6]). The association with papillomas is called Goltz-Gorlin syndrome [7, 8] and the associations with malign neoplasms are described in literature [9]. Furthermore there is a correlation with syndactyly and hemolytic anemias like spherocytosis [10] and leukemias [11]. To our knowledge this is the first report of a squamous cell carcinoma within the area of a symbrachydactyly.

## 2. Case Report

**Figure 1:**
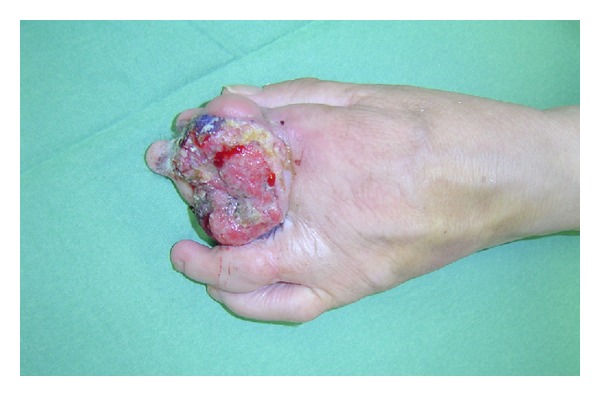
Preoperative status: 4 × 5 cm measuring, exulcerating and partly necrotic tumor.

In March 2005 a 68-year-old female patient presented to our outpatient clinic with a 4 × 5 cm sized exulcerating tumor at the left hand in the area of a congenital symbrachydactyly at the digiti II and III. (Figure 1). The tumor showed rapid progression and caused local pain. About four months prior to admission on our clinic the tumor was seen for the first time by a physician. The tumor was initially diagnosed as seborrheic keratosis, and for months it was treated with topical medication only. Additionally the patient was treated with antibiotics because of local inflammatory signs. The patient herself was in good general condition and had no B-symptoms and no increased lymph nodes were palpable.

**Figure 2:**
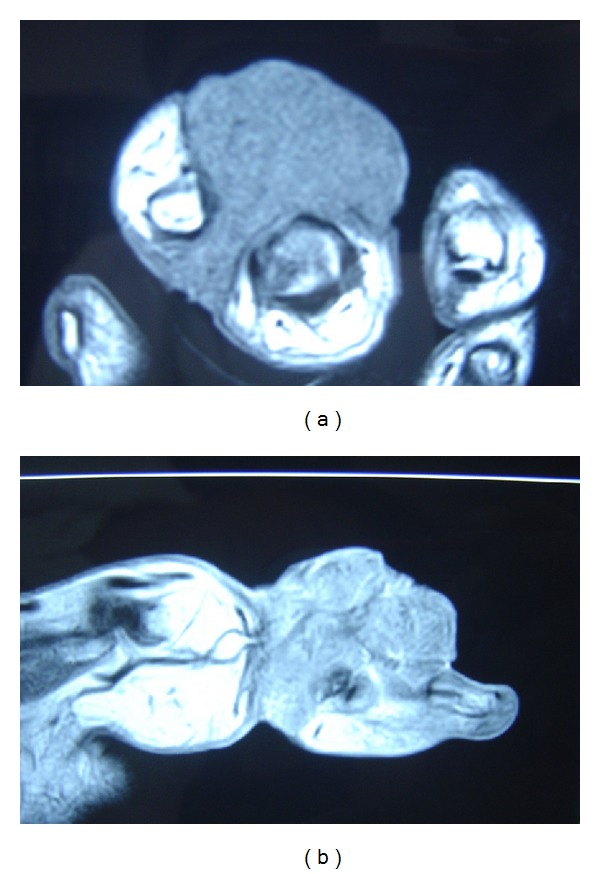
Preoperative MRI of the left hand shows an infiltrating soft-tissue mass with suspicion of malignancy (histiocytoma or fibrosarcoma) without osseous involvement.

An urgent biopsy of the tumor was performed, which confirmed our suspicion of a squamous cell carcinoma. Preoperative staging including MRI of the affected hand (Figures 2(a) and 2(b)), plain thoracic X-ray, abdominal ultrasound scan, and thoracoabdominal CT scan was performed. We diagnosed a soft-tissue tumor with evidence of malignancy, but with no evidence of bony infiltration nor intrathoracic or intra-abdominal metastasis. However we detected an enlarged sentinel lymph node in the left axilla. Further workup for abnormal lymph nodes showed two suspicious axillary enhancements.

Under curative intention we performed an amputation of the second and third ray of the left hand at the metacarpal level and additionally an axillar revision and lymph node dissection under general anesthesia.

The definite histological examination confirmed the preoperative suspicion of a squamous cell carcinoma, fortunately without affection of any lymph nodes (histological TNM staging: pT4 N0 M0 G1-2).

**Figure 3:**
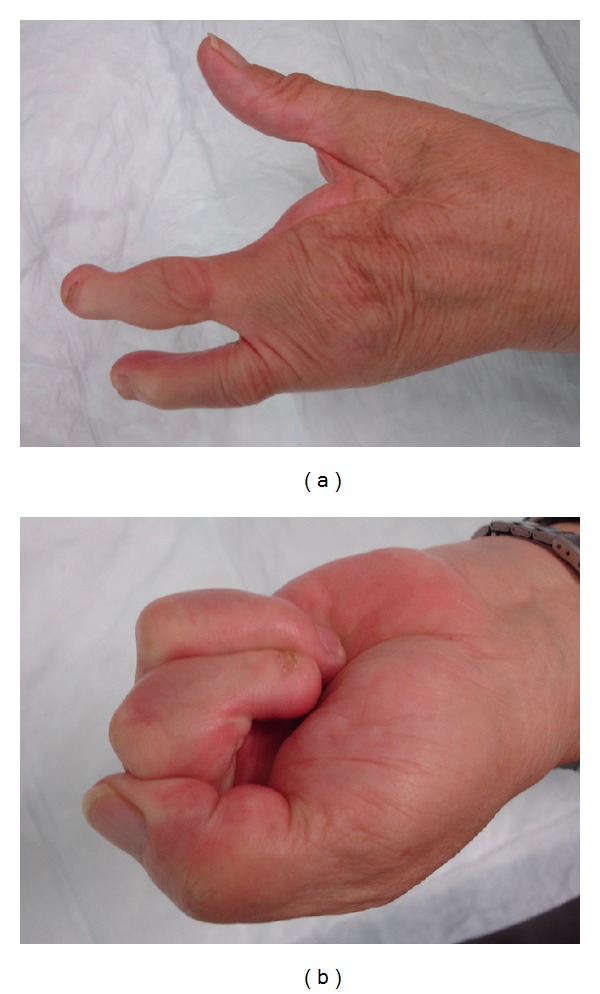
Postoperative result: excellent function of the hand.

The postoperative course was without complications except a recurring lymphoid seroma developing in the left axilla, which was successfully treated by repetitive aspiration. With intensive ergotherapy the patient reached an excellent functional result of the operated left hand (Figures 3(a) and 3(b)). 9 years after the surgery there was no clinical sign of any tumor recurrence. The patient could perform fine motor skills like sewing. The measurements of the motor function showed decreased strength of the operated left hand compared with the dominant right hand with finger pinch side-to-end (left 3.5 kg and right 7 kg), end-to-end (left 2 kg and right 5 kg), and hand grip (left 11 kg and right 26 kg).

## 3. Discussion

Malformations of the hand are rare conditions. The incidence of transverse failure of formation at the hand or finger level (including symbrachydactyly) is about 5.8 per 10.000 births [12]. Symbrachydactyly is often combined with other congenital malformations like dysplasias of the pectoral muscles (Poland's syndrome). In our patient we could not find any other congenital disorders and malformations, respectively.

Several genetic changes are known, which are considered to be responsible for congenital hand malformations. For example Schwabe et al. could show that mutation in the prodomain of CDMP1 leads to brachydactyly type C [13]. Recently was published the role of activating and deactivating mutations in the receptor interaction site of GDF5 in the development of symphalangism or brachydactyly type A2 [14].

Tumors of the hand are rare too. Within the malignant tumors of the hand squamous cell carcinoma is the most common, accounting for 75–90% of hand malignancies [1]. Well known is long-term ultraviolet light exposure as main risk factor for development of cutaneous squamous cell carcinoma [15]. Precancerous conditions like Morbus Bowen were also known to lead to squamous cell carcinoma. Furthermore overexpression and mutation of the tumor suppressor gene p53 is found in 90% of cutaneous squamous cell carcinoma lesions [16]. We could not find a reference that shows a common genetic predisposition to both symbrachydactyly and squamous cell carcinoma. And there is no evidence that the previously mentioned genetic hand dysplasia disorders weaken the skin resistance to UV light influence similar to Bloom's syndrome [2].

To our knowledge, this is the first description of a squamous cell carcinoma in the region of a symbrachydactyly. In the literature we could detect one case report of a malignant breast tumor in Poland's syndrome [9]. Another case report describes an infantile digital fibromatosis after a web construction that appeared at the skin graft edge in a girl, with a simple syndactyly [17].

It remains unclear whether our case is a coincidence of two rare independent diseases or there is a pathogenic link between the malformation and the tumor on a genetic level.
